# Supplementary material for: The Challenge of Developing a Test to Differentiate Actinobacillus pleuropneumoniae Serotypes 9 and 11
Source: Microorganisms. 2025 Jan 26;13(2):280. doi: 10.3390/microorganisms13020280 (PMC11858502; doi:10.3390/microorganisms13020280)
Supplement: Supplementary file 1 [file microorganisms-13-00280-s001.zip › Supplementary File Figure S1_alignment cpsF_submit.pdf]

Supplementary File.

Multiple Sequence Alignment of the *cpsF* locus from 12 Hungarian Isolates in Clinical Collection A (CCA)and reference strains of serotype 9 and 11. (UniproGene V50.0; Kalign2)

Figure S1a.

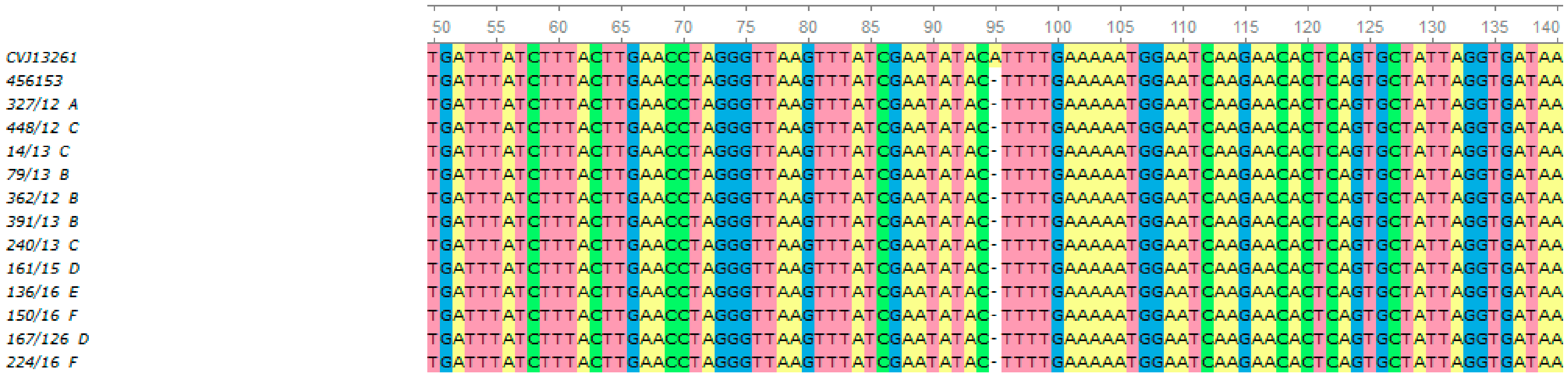

Considering as reference serotype 9 sequence (CVJ13261), all 12 Hungarian isolates had the previously mentioned deletion at position 95, identical to the serotype 11 reference strain.

Figure S1b.

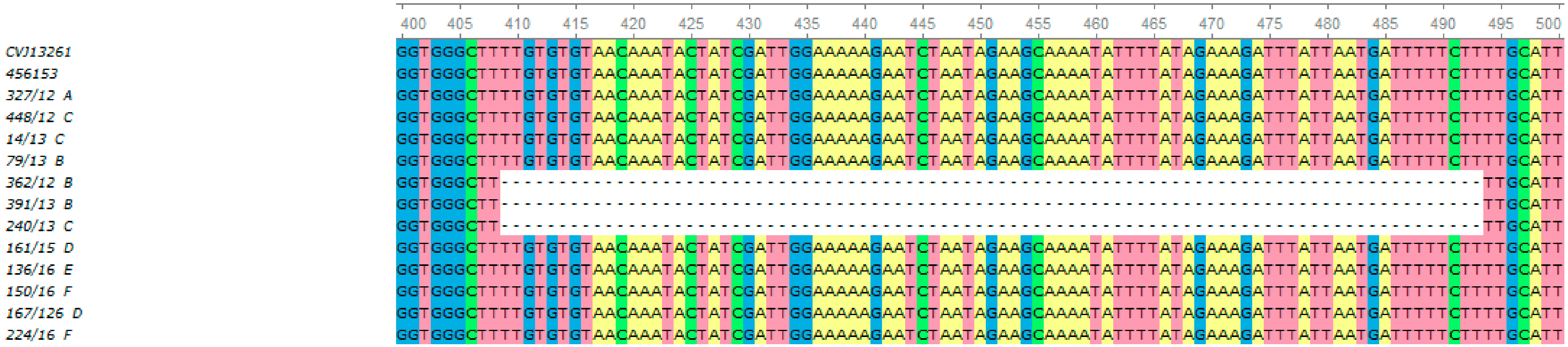

Isolates 362/12_B, 391/13_B and 240/13_C had an 85 bp deletion from positions 408 to 493, leading to a truncated ORF of 513 bp.

Figure S1c.

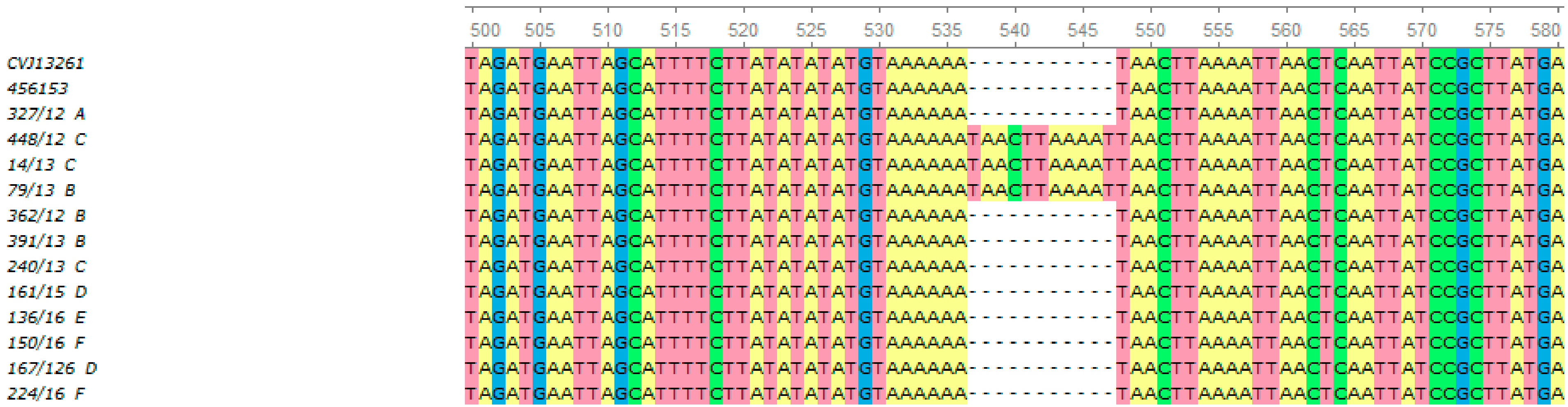

The isolates 448/12_C, 14/13_C, and 79/13_B had an 11 nt (TAACTTAAAAT) insertion at position 536, resulting in a shorter ORF of 651 bp.

Figure S1d.

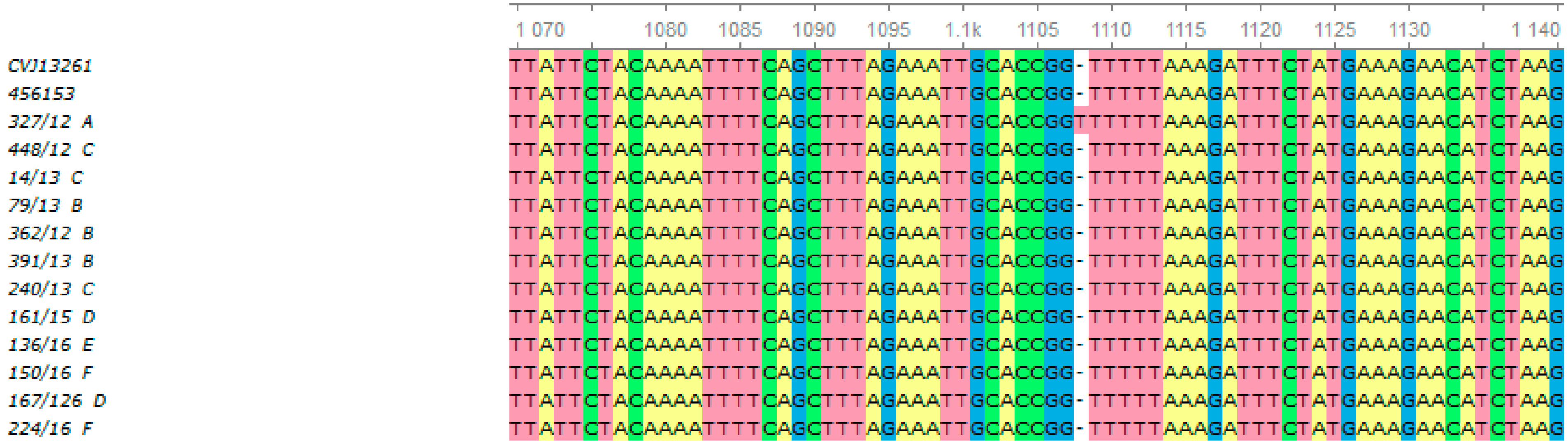

Isolate 327/12_A had an insertion of a thymine (T) nucleotide at position 1096, resulting in an alternative ORF of 1200 bp.
