# Supplementary material for: The Challenge of Developing a Test to Differentiate Actinobacillus pleuropneumoniae Serotypes 9 and 11
Source: Microorganisms. 2025 Jan 26;13(2):280. doi: 10.3390/microorganisms13020280 (PMC11858502; doi:10.3390/microorganisms13020280)
Supplement: Supplementary file 1 [file microorganisms-13-00280-s001.zip › Supplementary File Figure S2_OAg_submit.pdf]

Supplementary File.

Figure S2. Mutation events identified throughout the loci within the O-Ag operon of CCB isolates.

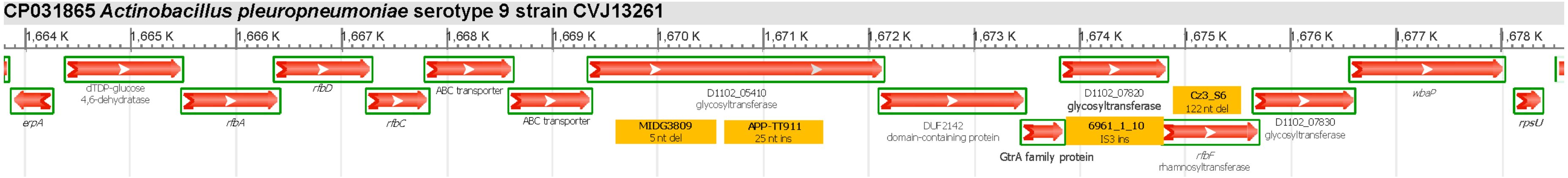

Four isolates, highlighted with yellow squares, have mutations that are predicted to impact on O-Ag expression.

Two isolates, MIDG3809 and APP-TT911, have mutations in the largest glycosyltransferase gene (locus tag D1102_05410, NCBI GenBank CP031865). MIDG3809 has a 5 bp deletion, while APP-TT911 has a 25 bp tandem duplication. Both mutations result in internal stop codons that are predicted to interfere with the expression of a functional protein.

The isolate 6961_1_10 contains multiple copies of insertion sequences with homology to IS3 family that disrupt several loci. These include the glycosyltransferase gene (locus tag D1102_07820; NCBI GenBank CP031865) located upstream of the *rfbF* gene.

All isolates, except Cz3_S6, have the *rfbF* gene identical in sequence to that found in the serotype 9 (CVJ13261) and 11 (456153) reference strains with rhamnosyltransferase function. In Cz3_S6 isolate, a 122 bp deletion has resulted in the fusion of the *rfbF* gene with the downstream D1102_07830 gene (NCBI GenBank CP031865) encoding N-glycosyltransferase, resulting in a potentially non-functional single ORF of 1659 bp.
